# RETURNED MISSING PERSONS WITH DEMENTIA: WHAT ROLE CAN FIRST RESPONDERS AND SERVICE PROVIDERS PLAY?

**DOI:** 10.1093/geroni/igac059.2470

**Published:** 2022-12-20

**Authors:** Christine Daum, Lauren McLennan, Elyse Letts, Cathy Conway, Lili Liu

**Affiliations:** University of Waterloo, Waterloo, Ontario, Canada; University of Waterloo, Waterloo, Ontario, Canada; University of Waterloo, Waterloo, Ontario, Canada; University of Waterloo, Toronto, Ontario, Canada; University of Waterloo, Waterloo, Ontario, Canada

## Abstract

The number of people living with dementia that wander and go missing is increasing. First responders and service providers play a role in the return of a missing person living with dementia. In the United Kingdom (UK), “return home interviews” are discussions between police and returned missing persons that offer support to the returned missing person to prevent repeat incidents. This study aims to explore and understand the role of first responders and service providers who follow-up with returned missing persons living with dementia. Eight service providers (e.g., social workers) and seven first responders (e.g., police officers) from Canada and the UK participated in online semi-structured interviews. Data were concurrently collected and analyzed using conventional content analysis. In the UK, police conduct “return home interviews” within 72 hours of the missing person’s return. Some charities conduct interviews with vulnerable populations to prevent repeat missing incidents by understanding the circumstances of the missing incident and connecting the person to community supports. In Canada, although follow-up with returned missing persons is not routine, some police units offer support to returned missing older adults. Government and community support organizations also offer supports to returned missing older adults such as referrals for in-home support, technologies, and vulnerable person registries. Service providers and first responders have an important role to play in the prevention of repeat missing incidents. Findings will contribute to the development of a Canadian practice guide for conducting interviews with returned missing persons living with dementia.

